# Real-time tracking of energy flow in cluster formation

**DOI:** 10.1038/s42004-025-01563-6

**Published:** 2025-05-29

**Authors:** Michael Stadlhofer, Bernhard Thaler, Pascal Heim, Josef Tiggesbäumker, Markus Koch

**Affiliations:** 1 Institute of Experimental Physic, Graz University of Technology, Graz, Austria; 2 Institute of Physics, University of Rostock, Rostock, Germany; 3 Department of Life, Light and Matter, University of Rostock, Rostock, Germany

**Keywords:** Photochemistry, Energy transfer, Chemical physics

## Abstract

Femtosecond time-resolved spectroscopy has shaped our understanding of light-matter interaction at the atomic level. However, the photoinduced formation of chemical bonds, especially for larger aggregates, has evaded observation due to difficulties to prepare reactants at well-defined initial conditions. Here, we overcome this hurdle by taking advantage of the exceptional solvation properties of superfluid helium, which allow us to stabilize atoms in a metastable, foam-like configuration with 10 Å interatomic distance. We apply photoexcitation with a femtosecond laser pulse to collapse such a dilute metastable aggregate of Mg atoms formed inside a nanometer-sized He droplet, and track cluster formation at a characteristic time of (450 ± 180) fs through photoionization with a time-delayed second pulse. We find that energy pooling collisions of electronically excited Mg atoms occur during cluster formation, leading to transient population of highly-excited Mg atoms, up to 3 eV above the excitation photon energy. Relaxation and conversion to nuclear kinetic energy drives cluster fragmentation and ejection of ionic fragments from the droplet. Our results demonstrate the potential of He droplets for bond formation studies, and for revealing involved energy- and charge transfer dynamics, like photon energy upconversion.

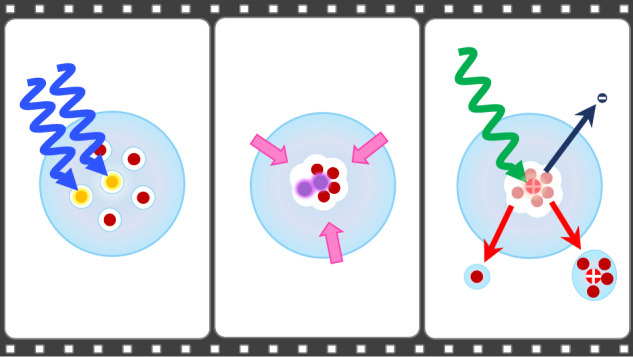

## Abstract (web summary)

Probing the photoinduced formation of chemical bonds in aggregates is challenging due to difficulties in reactant preparation under well-defined conditions. Here, the authors use helium droplets to stabilize Mg atoms in a foam-like configuration and track cluster formation using femtosecond time-resolved spectroscopy.

## Introduction

Chemical reactions essentially consist of breaking and forming of molecular bonds. The mechanistic understanding of photoinduced bond breaking has been particularly shaped through femtosecond pump–probe spectroscopy^1–3^. Real-time tracking of the electronic and nuclear structure has provided insight into various processes accompanying photodissociation, such as curve crossings^4,5^, predissociation^6^ conical intersections^7^, or electronic relaxation^8^.

Photoassociation is used to form molecules in ultracold atomic clouds^9,10^, where the binding energy is dissipated into the light field^11^. In the time–domain, however, photoassociation has largely escaped observation so far, in particular for larger systems, due to difficulties in preparing the reactants in a well-defined initial geometry. This limitation could be overcome only in a few situations: In gas phase, where the broad distribution of impact parameters completely blurs the time resolution, bond formation could only be observed for selected dimer molecules through Franck–Condon filtering based on the resonance condition for laser excitation^12–14^. In a few cases, cold bimolecular van der Waals complexes^15–18^, and anionic clusters^19^, provide favorable initial conditions to study bond formation. In solution, a network of species separated by well-defined distances are readily achieved, however, inhomogeneous broadening prevents observation of individual states and structural dynamics can only be inferred by x-ray scattering^20,21^. Despite these versatile approaches, the formation of clusters larger than dimer molecules and bimolecular aggregates has not been experimentally realized so far.

The endeavor to combine the advantages of the previous approaches—homogeneous distances in solution, low environmental perturbation for well-defined resonances, and low temperatures in gas phase—leads us to the application of helium nanodroplets (He_N_) as a cryogenic solvent. Helium nanodroplets have routinely been used for the synthesis and investigation of atomic and molecular aggregates, since they provide a high degree of control in the aggregation process, efficient cooling to 0.37 K, and enable measurements with low matrix effects compared to other noble gas environments^22–25^. Here, we show that the unique solvation properties of superfluid He can be used to prepare well-defined initial conditions for the time-resolved observation of bond formation of multiple reactants. This demonstration builds on recent observations that atoms solvated in He_N_ can arrange in metastable configurations at nanometer distance, enabled by the ultracold He solvent^26–32^. For two Mg atoms inside a He_N_, density functional theory simulations predict such a metastable configuration at 9.5 Å interatomic distance, instead of the formation of a Mg_2_ molecule^33^. This dilute configuration is enabled through the accumulation of helium density between two Mg atoms, and referred to as “foam”^33^ or “quantum gel”^34,35^. We note that this prediction has been subject to discussion, since path integral Monte Carlo simulations of two and three Mg atoms inside He_N_ find no evidence for stabilization in a metastable configuration, but only equilibration to the strongly bound dimer and trimer^36^.

**Fig. 1:**
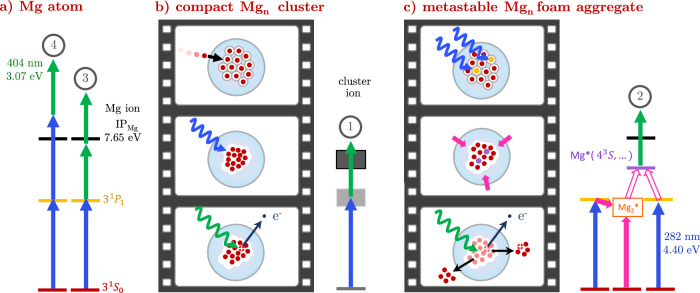
Sketch of the photoinduced dynamics of single Mg atoms, compact Mg_n_ cluster and metastable Mg_n_ foam inside He_N_. The corresponding energy level diagrams depict pump (blue)-probe (green) photoionization and different ionization pathways are labeled with (1) to (4), correspondingly to the photoelectron bands in Fig. 2. **a** Three-photon ionization channels of Mg atoms. **b** Formation of compact Mg_n_ cluster through collision of an energetic Mg atom with the foam-like aggregate during the pickup process^43^ and subsequent pump-probe photoionization. **c** Photoexcitation of Mg atoms within the Mg_n_ foam triggers the transition to a compact cluster. Energy pooling (magenta arrows) leads to population of highly excited Mg levels and fragmentation.

Here, we present an investigation of the dynamical response of Mg_n_ aggregates inside He_N_ to photoexcitation with femtosecond photoelectron and -ion spectroscopy^2,3^. This approach has recently proven successful inside He_N_ for the observation of electronic^37,38^ and nuclear dynamics^39,40^. We obtain time-resolved photoelectron spectra (TRPES) containing two different dynamic signatures, indicating that Mg_n_ aggregates exist in two different configurations inside He_N_. An immediate signal rise followed by a fast *τ*_1_ = (380 ± 70) fs decay is characteristic for van der Waals clusters, where primary processes are electronic dynamics (Fig. 1b). In addition to this swiftly responding compact clusters, the TRPES also contains a PE band with delayed signal rise peaking 1.2 ps after photoexcitation and a much slower *τ*_2_ = (4.0 ± 0.9) ps decay. We interpret this slower response to photoexcitation as a signature for nuclear dynamics involved in the transition leading from the predicted foam-like Mg_n_ configuration to a dense cluster (Fig. 1c). This transient signal reveals insight into the energy flow and nuclear dynamics during cluster formation.

## Results

**Fig. 2:**
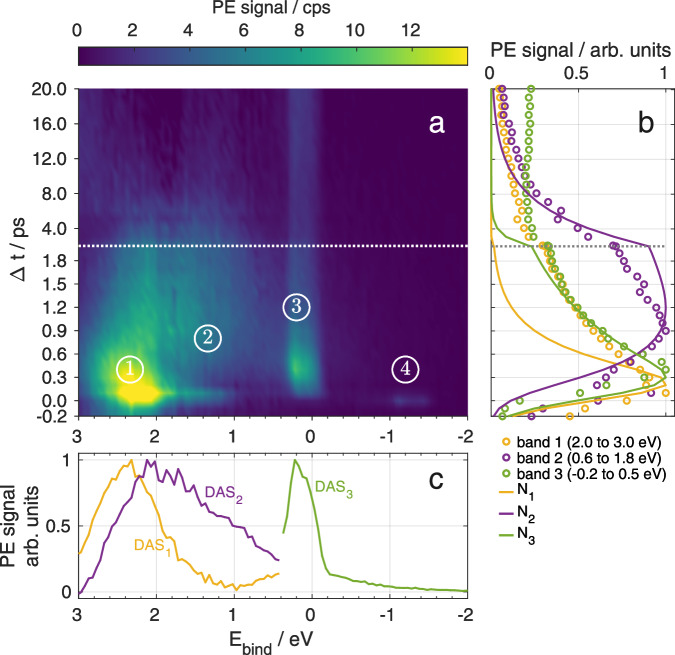
Time-resolved photoelectron spectrum and global fitting analysis of Mg_n_ aggregates embedded in He_N_. **a** Pseudocolor plot showing the transient electron yield as function of binding energy. Four distinct bands are marked (1) to (4). **b** Comparison of energy-integrated PE transients (see legend) to decay functions N_1,2,3_ from global fitting. **c** Decay associated spectra DAS_1,2,3_ obtained from global fitting. Note that below 2 ps, the spectra feature a higher temporal resolution.

The time-resolved photoelectron spectrum is shown in Fig. 2, depicted as a function of the electron binding energy *E*_bind_ (*E*_bind_ = *hν*_probe_ − *E*_el.kin_, where *E*_el.kin_ is the measured electron kinetic energy). We first allocate the observed photoelectron bands to pump–probe ionization pathways based on energetic considerations of the 282 nm (4.40 eV photon energy) pump pulse and the 404 nm (3.07 eV) probe pulse. The temporal development of these bands, obtained from a global fitting analysis, reveals population transfers dynamics triggered by photoexcitation. Examination of the time-resolved ion yields and, in particular, the correlation of ion fragments to the photoelelctron bands gives information about the accompanying nuclear dynamics.

### Assignment of photoelectron bands

Inspection of Fig. 2a reveals four distinct bands. Band (1) between 2 and 3 eV rises instantaneously and shows a fast decay (Fig. 2b, yellow circles). Band (2) between 1 and 2 eV shows initially a cross correlation feature, followed by a moderate signal rise peaking at 1.2 ps and a slower decay than band (1) (Fig. 2b, purple circles). The assignment of bands (1) and (2) in Fig. 2 is difficult due to their overlap in time and energy. Both bands are related to states with binding energies lower than that of the photoexcited bare-atom 3^1^P_1_ state (3.30 eV binding energy), given the lower probe photon energy of only 3.07 eV. Bands (3) and (4) can be assigned to two-photon ionization of the excited 3^1^P_1_ Mg state (see Fig. 1a). Band (3) extends from −0.17 eV to 0.5 eV with a fast rise and slower decay, followed by a second slow rise after 10 ps (see Fig. 2b). This band represents the transient population of 3^1^P_1_ Mg state, ionized by two probe photons. Note that the increased peak width is common for photoionization of atoms inside He droplets^37^. The brief appearance of band (4) around *t*_0_ is characteristic for a cross-correlation peak: With a binding energy slightly below –1 eV we assign this band to 3^1^P_1_ ionization with one pump and one probe photon.

### Global fitting analysis to retrieve population transfer dynamics

The spectral and temporal overlap of bands (1) and (2) in the time-resolved photoelectron spectrum (Fig. 2) poses a challenge for a quantitative analysis. In order to decode the different contributions, the spectrum is analyzed by applying a global fitting procedure^41,42^, i.e., species with different transient behavior contributing to the spectrum are identified by extracting the respective decay associated spectra, DAS(*E*), and the associated transient decay functions, *N*(*t*). Since the photoelectron bands in Fig. 2 differ in their signal rise behavior, we use two different types of decay functions: An instantaneous signal rise is modeled by a directly excited state followed by population decay (a Gaussian function convoluted with an exponential decay, as described in detail in Supplementary B). A delayed signal rise is modeled by assuming sequential population transfer from an initially excited state (which is not necessarily detected) into the state yielding the photoelectron signal of the observed band. This function accounts for an exponential signal rise followed by an exponential decay, with two different characteristic time constants.

Taking into account photoelectron-band assignments already made, one can simplify the fit process: Since band (3) originates from a different ionization process than bands (1) and (2) and since there is no overlap of band (3) with bands (1) and (2), one can split the time-resolved PE data into two energy domains at 0.42 eV binding energy. The low binding energy region, containing the 3^1^P_1_ atom band (3), is modeled by DAS_3_ (Fig. 2c, green line). The 3^1^P_1_ population appears instantly (Fig. 2b, green line), suggesting direct excitation by the pump pulse, and then decays with a time constant of *τ*_3_ = (1.1 ± 0.1) ps.

Relevant parameters of the decay functions are listed in Table 1 for better comparability. From this transient signal, one can deduce the temporal pump-probe overlap (time zero, *t*_0_) and the temporal instrument response function duration *σ*. Inspection of the low binding energy region in Fig. 2 shows that the signal rises towards long delay times, which is not represented by the fit function. We account for this deviation by introducing an additional background, as described in Supplementary B. Furthermore, the pump–probe cross-correlation signal of band (4) is determined to be (45 ± 3) fs in a separate measurement of the total electron yield around time zero. This cross-correlation signal, together with *t*_0_ and *σ*, is kept constant in the remaining global fitting process.

**Table 1:** Decay parameters

parameter	value / fs	feature
time zero, *t*_0_	45 ± 4	all
temporal instrument response, *σ*	170 ± 15	all
*N*_1_ decay time, *τ*_1_	380 ± 70	DAS_1_
*N*_2_ rise time, ${\tau }_{2}^{rise}$	450 ± 180	DAS_2_
*N*_2_ decay time, *τ*_2_	4000 ± 900	DAS_2_
*N*_3_ decay time, *τ*_3_	1090 ± 90	DAS_3_

Relevant parameters of the three decay functions as obtained from the global fitting procedure (see Supplementary B for formulas of the decay functions). Uncertainties represent a confidence level of 95%. In addition to the parameter and its value, the rightmost column indicates the DAS corresponding to the decay function.

In the high binding-energy domain two distinct populations, represented by different decay associated spectra and different decay functions, can be expected. In agreement with this assumption, DAS_1_ with a peak at 2.5 eV (Fig. 2c, yellow line) and the broader DAS_2_ peaking at 2 eV (purple line) can be identified, together with two corresponding decay functions. The transient population *N*_1_ rises quickly to a maximum at  ~250 fs, followed by a rapid decay with a characteristic time of *τ*_1_ = (380 ± 70) fs (Fig. 2b, yellow line). Note that the integrated 2.0–3.0 eV signal (yellow circles) deviates from the *N*_1_ transient (yellow line) because there is a significant contribution from the slower *N*_2_ (DAS_2_) signal in this energy interval. *N*_2_ features a delayed onset with respect to *N*_1_ with a rise time of ${\tau }_{2}^{{{{\rm{rise}}}}}=(450\pm 180)$ fs leading to a maximum at 1.2 ps and also a slower decay time constant of *τ*_2_ = (4.0 ± 0.9) ps (Fig. 2b, purple line).

### Interpretation of DAS populations

Although the similarity of the band (1) decay time and the band (2) rise time might suggest sequential population transfer from states correponding to band (1) to states corresponding to band (2), we do not consider this option because band (2) appears at lower binding energy (energetically higher states) than band (1). For this conclusion it is important that the ionization process leading to bands (1) and (2) proceeds with the same number of probe photons, which we have verified through intensity dependence measurements. The different transient behavior of bands (1) and (2) thus indicates the presence of two different species. The sudden appearance of band (1) represents direct electronic excitation, presumably of compact Mg_n_ clusters. These compact clusters, which are formed through collision of an energetic Mg atom with the foam-like aggregate during the pickup process, have a reduced electron binding energy compared to Mg atoms^43^ and can thus be ionized by the probe pulse immediately after excitation (see Fig. 1b).

The picosecond delay in the onset of band (2), in contrast, suggests that nuclear dynamics are involved, which are caused by the population of an undetected excited state. A possible origin would be the presence of a previously suggested foam-like Mg_n_ configuration, in combination with a transition to a compact Mg_n_ cluster triggered through 3^1^P_1_ ← 3^1^S_0_ photoexcitaiton by the pump pulse (see Fig. 1c)^30,32,33^. Given the 3^1^P_1_ excited state binding energy of 3.30 eV, it is remarkable that DAS_2_ indicates transient population within the whole detection window given by the 3.07 eV photon energy of the probe pulse. In this scenario, the occupation of these highly excited states is caused by the transition of Mg_n_ aggregates from a dilute to a compact configuration.

Testing the hypothetical assignment of band (1) to a compact Mg_n_ cluster and band (2) to a forming Mg_n_ cluster, the dependence of the photoelectron signal on the Mg doping level (see Supplementary Fig. A.1) was investigated. At high doping, band (2) decreases and band (1) increases in relative strength, consistent with earlier studies of the spontaneous collapse of the dilute Mg aggregate at high Mg doping concentrations^43^.

Direct photoexcitation of Mg atoms to states with such low binding energy, can be excluded since two-photon excitation leads to ionization (4.4 eV photon energy, 7.65 eV ionization energy^44^). Also, combined excitation with one pump and one probe photon can be excluded since this can only occur when pump and probe pulse overlap.

### Transient photoion signal

**Fig. 3:**
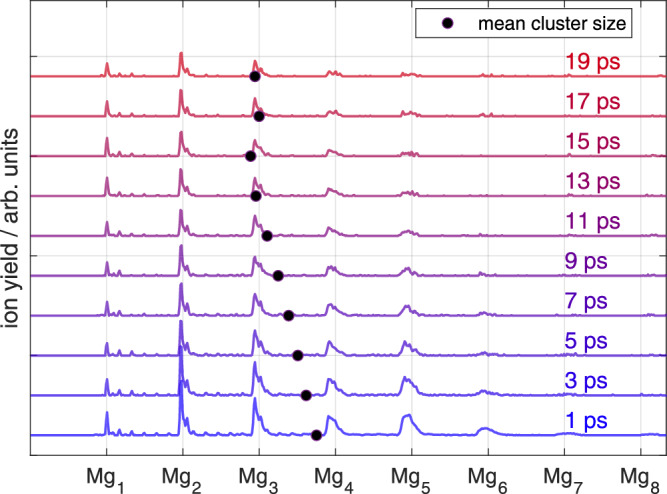
Ion mass spectra for different pump–probe time delays. The mass spectra represent the transient fragment distribution of ${{{{\rm{Mg}}}}}_{{{{\rm{n}}}}}^{+}$ cluster ions formed after photoexcitation and probe ionization. The spectra are area normalized and vertically offset according to the time-delay, indicated for each spectrum on the right side. Both isolated ${{{{\rm{Mg}}}}}_{{{{\rm{n}}}}}^{+}$ clusters and ${{{{\rm{Mg}}}}}_{{{{\rm{n}}}}}^{+}$-He_m_ snowballs are present in the mass spectra. The spectra were obtained by averaging the ion mass spectra in 2 ps time intervals and the mean cluster size is indicated as a black dot.

A more direct insight into the nuclear dynamics and in particular the fragmentation behavior can be gained from the ion signals. Ions expelled from the droplet show up at time delays larger than *Δt* ≥ 1 ps. Figure 3 shows pump-probe ion spectra recorded at selected time-delays. The spectra consist of peaks corresponding to ${{{{\rm{Mg}}}}}_{{{{\rm{n}}}}}^{+}$ (*n* = 1–8) clusters and ${{{{\rm{Mg}}}}}_{{{{\rm{n}}}}}^{+}{{{{\rm{He}}}}}_{{{{\rm{m}}}}}$ snowballs.

The pump-probe spectra show ion signals only up to ${{{{\rm{Mg}}}}}_{8}^{+}$, while in the probe-only signal, masses as high as ${{{{\rm{Mg}}}}}_{12}^{+}$ appear. The average cluster size, taken as a measure and indicated by a black dot in Fig. 3 for each spectrum, decreases from 3.7 to 2.8 with increasing delay. Neglecting that the ionization probability may change with cluster size, this down-shift reflects the general trend that an increasing amount of energy is transferred to nuclear degrees of freedom with time.

### Correlated electron–ion detection to identify fragmentation dynamics

To further test the assignment of TRPES band (1) to compact Mg_n_ clusters and band (2) to foam-like aggregates, we examine correlations between photoelectrons and ions through covariance detection^45–47^. The assignment of ion fragments to each of the bands is of particular interest. This detection method has been applied to gas-phase molecules, where it allows to distinguish between different photochemical reaction pathways based on the ionic products^48–50^. For photoionization inside a helium droplet, the electrostrictive attraction prevents ion detachment from the droplet, except for situations where the ions gain sufficient kinetic energy through photodissociation^51,52^, or Coulomb explosion^40,53^. In our case, we will see that the processes behind photoelectron band (1) and (2) differ significantly in the probability to yield ${{{{\rm{Mg}}}}}_{{{{\rm{n}}}}}^{+}$ ion ejection from the droplet.

**Fig. 4:**
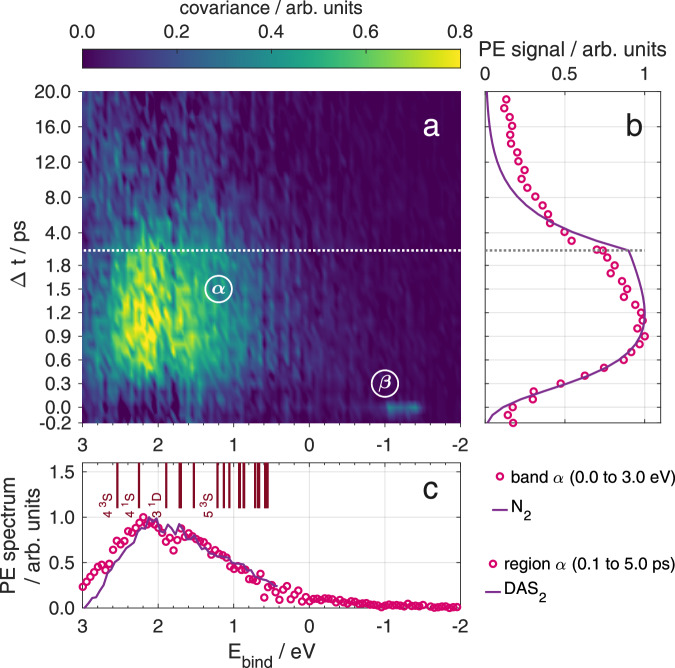
Time-resolved photoelectron-photoion covariance spectrum of Mg_*n*_ aggregates inside He_N_. **a** Pseudocolor plot of TRPES correlated to ion masses between 16 and 300 u. Two energy bands (*α*) and (*β*) are marked. **b** Comparison of energy-integrated covariance band (*α*) to the time-dependent decay function *N*_2_ of DAS_2_. **c** Comparison of the time-integrated covariance band (*α*) to DAS_2_. Red vertical lines indicate highly excited Mg atom states^44^.

Figure 4 shows the time-resolved PE spectra detected in covariance with ${{{{\rm{Mg}}}}}_{{{{\rm{n}}}}}^{+}$ (*n* = 1 − 12). In contrast to the transient PE spectrum of all electrons in Fig. 2, only two bands show up: A broad band (*α*) that extends from 0.5 to 3 eV with a slow rise and fall time, and the cross-correlation band (*β*) originating from three-photon ionization of gas-phase Mg atoms [corresponding to band (4) in Fig. 2]. We compare covariance band (*α*) to the global fit results of TRPES band (2), separately in the spectral domain (Fig. 4c) and in the temporal domain (Fig. 4b). This comparison reveals good agreement in both domains, which is a strong indicator that both observables reflect the same underlying photoinduced process, especially under consideration of the difference of the experimental methods. The fact that only band (2) of the TRPES is apparent in the covariance spectrum, while band (1) is missing, shows that only the photoexcitation process represented by band (2) leads to ejection of Mg^+^ ions. In contrast to the dilute aggregate, photoexcitation of compact Mg_n_ clusters (DAS_1_) leads to immediate promotion of electrons into the detection window for one-photon ionization of the probe pulse [band (1) in Fig. 2]. Fragmentation caused by energy conversion to nuclear motion appears much less pronounced for the compact clusters so that ion ejection from the droplet is prevented, eliminating band (1) from the covariance spectrum (Fig. 4). This finding further supports our hypothetical assignment that the two bands correspond to different species with different photoexcitation dynamics: compact Mg_n_ clusters associated with DAS_1_ and the foam-like configuration associated with DAS_2_ and covariance band (*α*).

**Fig. 5:**
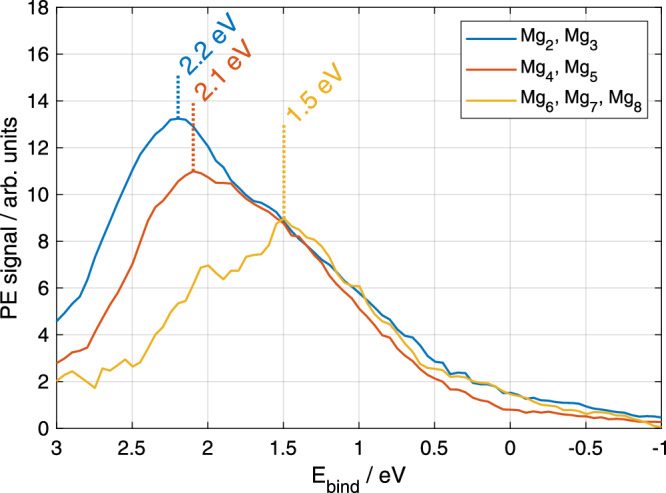
Time-integrated photoelectron spectra correlated with different ${{{{\rm{Mg}}}}}_{{{{\rm{n}}}}}^{+}{{{{\rm{He}}}}}_{{{{\rm{m}}}}}$ ion complexes. The photoelectron spectra were integrated in a time-window of 0.1 ps to 5 ps. The sizes of ${{{{\rm{Mg}}}}}_{{{{\rm{n}}}}}^{+}{{{{\rm{He}}}}}_{{{{\rm{m}}}}}$ complexes are indicated in the legend. For the presentation, a moving mean with a width of 0.4 eV is applied.

An assignment of the electron signal shown in Fig. 4 to specific cluster sizes provides additional insight into the fragmentation process. To this end, Fig. 5 shows time-integrated (0.1–5 ps) electron spectra correlated to the ion complexes ${{{{\rm{Mg}}}}}_{2,3}^{+}{{{{\rm{He}}}}}_{{{{\rm{m}}}}}$, ${{{{\rm{Mg}}}}}_{4,5}^{+}{{{{\rm{He}}}}}_{{{{\rm{m}}}}}$ and ${{{{\rm{Mg}}}}}_{6,7,8}^{+}{{{{\rm{He}}}}}_{{{{\rm{m}}}}}$, with *m* = 0–5. The most likely energy, marked by vertical lines in Fig. 5, decreases from 2.2 eV for ${{{{\rm{Mg}}}}}_{2,3}^{+}{{{{\rm{He}}}}}_{{{{\rm{m}}}}}$ to 1.5 eV for ${{{{\rm{Mg}}}}}_{6,7,8}^{+}{{{{\rm{He}}}}}_{{{{\rm{m}}}}}$. This development shows that electronic relaxation to energetically lower states yields smaller fragments, as more electronic energy is converted to nuclear kinetic energy.

## Discussion

The combination of global fitting analysis of PE spectra and electron-ion covariance detection provides evidence that He droplets enable the formation of two Mg_n_ configurations. These two configurations, represented by bands (1) and (2) in Fig. 2, show distinctively different photoexcitation responses, which is identified through the transient population of states with binding energies below 3.07 eV, corresponding to the one-photon detection window of the probe-pulse. We now discuss the energetics of the excitation processes leading to the population of these low-binding-energy states. Concerning band (1), compact Mg_n_ clusters have a lower ionization potential than the single Mg atoms and since calculated absorption spectra of small Mg_n_ clusters^54,55^ overlap with our pump photon energy, efficient pump-probe photoionization can be expected (Fig. 1c). The energetics leading to band (2) are less obvious: The transition from foam-like aggregate to compact cluster is triggered by the 3^1^P_1_ atom excitation, which has a binding energy of 3.30 eV and thus lies outside the one-photon detection window. The highly excited state population of band (2) in the 0–3 eV binding energy range appears delayed, with a rise time of ${\tau }_{2}^{{{{\rm{rise}}}}}=(450\pm 180)$ fs (see Fig. 2 and band (*α*) in Fig. 4). This raises the question about the processes leading to population of these low-binding-energy states up to the ionization continuum. A further question that will be discussed in the following is the different fragmentation behavior of the two Mg_n_ configurations in terms of ion ejection from the droplet.

### Energy-pooling reaction

The observed population of excited states above the pump photon energy is reminiscent of energy pooling reactions, observed in mixtures of metal vapor and a noble gas^56^. For Mg, 457.1 nm excitation of two atoms from the 3^1^S_0_ ground state to the 3^3^P_1_ excited state at 4.94 eV binding energy in a He buffer gas environment leads to population of the higher states 3^1^P_1_ and 4^3^S_1_ at 3.30 eV and 2.54 eV binding energy, respectively^57^.

In our experiment, aggregates of cold Mg atoms are photoactivated in a dilute configuration with a Mg–Mg distance of 9.5 Å^33^. The excitation energy for the single–atom 3^1^P_1_ ← 3^1^S_0_ transition in this environment is slightly blue-shifted to 4.40 eV photon energy (282.5 nm), relative to the bare-atom transition at 4.35 eV (285.5 nm)^44^. The dynamics in the He solvation shell in response to the photoexcitation process^37^ leads to collapse of the dilute configuration and formation of a compact Mg_n_ cluster. Interaction of ground-state and electronically excited Mg atoms leads to population of various states above the initially excited 3^1^P_1_ state, as shown by the photoelectron signal in Fig. 4c. While population of these states cannot result from a two-photon excitation process (see above), energy transfer through collisions of two excited 3^1^P_1_ atoms can populate all Mg states up to the ionization potential. We thus propose a similar energy pooling reaction, based on the excited-state potential energy curves of Mg_2_^58,59^. The reaction starts with the He-mediated formation of excited ${{{{\rm{Mg}}}}}_{2}^{* }$ molecules (see also Fig. 1c):

$${{{{\mathrm{Mg}}}}}(3^1{{{{\mathrm{P}}}}}_1) + {{{{\mathrm{Mg}}}}}(3^1{{{{\mathrm{S}}}}}_0) + {{{{\mathrm{He}}}}} \rightarrow {{{{\mathrm{Mg}}}}}_2^*({\scriptstyle{1}\atop} \!{{{\Sigma}}}^+_{{{{\mathrm{u}}}}}, {\scriptstyle{1}\atop} \!{{{\Pi}}}_{{{{\mathrm{g}}}}}) + {{{{\mathrm{He}}}}}$$

Collision of ${{{{\rm{Mg}}}}}_{2}^{* }$ with another excited Mg^*^ atom leads to population of the highly-excited states:

$${{{{\mathrm{Mg}}}}}_2^*({\scriptstyle{1}\atop} \!{{{\Sigma}}}^+_{{{{\mathrm{u}}}}}, {\scriptstyle{1}\atop} \!{{{\Pi}}}_{{{{\mathrm{g}}}}}) + {{{{\mathrm{Mg}}}}}(3^1{{{{\mathrm{P}}}}}_1) \rightarrow {{{{\mathrm{Mg}}}}}(4^3{{{{\mathrm{S}}}}}, 4^1{{{{\mathrm{S}}}}}, 3^1{{{{\mathrm{D}}}}}, ...) + 2 {{{{\mathrm{Mg}}}}}(3^1{{{{\mathrm{S}}}}}_{0})$$

In the covariance spectrum shown in Fig. 4, the photoelectron band spanning from 0 to 3 eV binding energy reveals the corresponding transient population distribution. On top of Fig. 4c, electronically excited states of Mg atoms are indicated for comparison. Since Mg_n_ clusters up to *n* ~20 atoms exhibit non-metallic van der Waals-type bonding^60^, we refer to atomic states, which are, however, not resolved in the PE spectrum due to environmental broadening and laser bandwidth.

The proposed energy pooling reaction requires at least two excited Mg atoms in the foam-like aggregate. One can estimate the excitation probability of one Mg atom to be *p*_1_ = 0.81 ± 0.15, based on the photon absorption cross section and photon density, including experimental uncertainties (see Supplementary C). Excitation of at least two Mg atoms is therefore quite likely.

### Cluster formation and fragmentation dynamics

The characteristic rise time of the transient photoelectron signal represents the highly-excited state population [*N*_2_ in Fig. 2b and band (*α*) in Fig. 4b]. This assignment is based on the correlated detection of electrons and ions: Only ions originating from ionization processes yielding band (*α*) [band (2)] electrons obtain sufficient kinetic energy to escape from the attractive solvation potential of the He droplet. Therefore, the rise of the ion-correlated photoelectron spectrum represents the transition from a foam-like Mg aggregate to a compact cluster. From parameters of the global fit population *N*_2_, a time constant of ${\tau }_{2}^{{{{\rm{rise}}}}}=(450\pm 180)$ fs for cluster formation is determined. This value agrees with the characteristic time-constant of 350 fs obtained by pump–probe strong-field ionization, which was proposed to represent the collapse of the dilute foam-like configuration^30^.

Concerning nuclear dynamics, including fragmentation of the Mg aggregate and acceleration of the fragments, it is important to realize that electronic energy can be converted into kinetic energy in each step of the energy pooling reaction. Initially, He atoms carry away some of the 4.4 eV photon energy stored in Mg^*^ in order to form the ${{{{\rm{Mg}}}}}_{2}^{* }$ bond. In step two, the ${{{{\rm{Mg}}}}}_{2}^{* }$–Mg^*^ collision can lead to the population of various higher excited Mg^*^ states (4^3^S, 4^1^S, 3^1^D, ...). The difference in excitation energies of reactants (8.7 eV) and products is thus converted into Mg kinetic energy, ranging from ≤3.6 eV for population of the 4^3^S state to ≤1.9 eV for population of states close to the IP (the ”≤” accounts for the kinetic energy of the He atoms)^44^. The released kinetic energy will increase the cluster temperature and, since the amount of energy is comparable to the binding energy of small Mg_n_ clusters^61^, fragmentation and ejection from the droplets are expected. Figure 5 supports this assumption by showing that the population of lower electronic states is correlated with smaller clusters. This is in line with previous observations, where liberation of ions from the droplet (overcoming the solvation energy) is only possible through a release of kinetic energy^51^. Note, however, that the ionization potentials decreases with cluster size^43^.

The covariance measurements also reveal that the ion–to–electron ratio decreases from 52% for gas-phase Mg atoms (a characteristic value of our covariance spectrometer) to  ~15% for Mg_n_–He_N_. This shows that the energy release is only in one out of three cases sufficient for liberation of the fragment ion from the solvation energy of the He droplet.

### Relaxation of excited clusters through electron–phonon interaction

The decaying character of the TRPES signal in Fig. 2, observed for both compact [band (1)] and foam-like aggregates [band (2) in Fig. 2 and (*α*) in Fig. 4], indicate on fast electronic relaxation. The characteristic decay time constant of the compact Mg_n_ cluster is *τ*_1_ = (380 ± 70) fs. Electronically excited metal clusters typically relax via electron–electron interaction on a time scale of less than  ~100 fs^2,62,63^. Our observation of a slower decay thus supports the assumption of a non-metallic complex^64^, in line with recent studies showing small magnesium clusters (*n* ≤ 18) are not metallic^65,66^. The electronic relaxation in van der Waals clusters proceeds through non-adiabatic transitions to lower electronic states with vibrational excitation. Electron–phonon coupling thus leads to electronically relaxed but vibrationally hot clusters.

For the Mg_n_ clusters formed from the foam-like configuration, the photoelectron signal decay reveals a ten times longer time constant of *τ*_2_ = (4.0 ± 0.9) ps, compared to the compact clusters. This further points at the non-conductive van der Waals nature of Mg_n_ clusters. The significantly slower decay might be rooted in the excitation of higher electronic states due to energy pooling, or a reduced electron–to–phonon energy transfer because the formed clusters are hot and potentially reduced in size due to fragmentation. Also, clusters (fragments) ejected from the droplet loose contact to the thermal bath, keeping them vibrationally excited for longer times. The transformation of electronic to vibrational energy within  ~10 ps leads to a reduction of the cluster fragment size, as depicted in the time-resolved mass spectra in Fig. 3. Electronic relaxation of the formed cluster also manifests as increase of the 3^1^P_1_ population with a characteristic time of ${\tau }_{BG}^{low}=3.3\,{{{\rm{ps}}}}$ (see band (3) in Fig. 2 and Supplementary B). The increasing 3^1^P_1_ population establishes agreement with the steady-state photoelectron spectra reported for dilute Mg_n_ ensembles in He_N_^27^. In photoemission experiments using nanosecond laser pulses, a strong signal from 3^1^P_1_ and a relatively weak signal in the region above at 1–2 eV binding energy is observed. Considering that excitation and ionization occurs with two photons within a 10 ns time window, in combination with the picosecond lifetime of higher excited states and the increasing 3^1^P_1_ population, our time-domain observations are in agreement with the steady state results.

## Conclusions

Femtosecond time-resolved photoelectron and -ion spectroscopy and a subsequent global fitting analysis has been used to study the photo-induced dynamics of small magnesium clusters with emphasis on foam-like complexes. Photoexcitation of this metastable Mg_n_ configuration leads to the contraction of the aggregate on a picosecond timescale. This contraction initiates the transient population of highly excited Mg states through energy pooling, as well as pronounced nuclear dynamics, which can clearly be resolved and distinguished from the compact Mg cluster response by inspecting the transient photoelectron signals.

The crucial prerequisite for this observation is the stabilization of Mg atoms at nanometer interatomic distance inside superfluid helium. First attempts to simulate these exceptional solvation properties of Mg atoms in He_N_ have led to contradictory results: While static DFT simulations predicted stable separation of two Mg atoms at 9.5 Å distance in He^33^, path integral Monte Carlo simulations found only equilibration to strongly bound Mg_2_ and Mg_3_ molecules^36^. Clarification of this discrepancy with frequency-domain spectroscopy might be challenging because the absorption spectra of small Mg_n_ clusters are predicted to show a small dependence on the cluster size^54,55^, with many of them absorbing at the 4.4 eV transition of the metastable configuration^32^. A very recent beam depletion study of Mg clusters in He droplets only shows a weak depletion signal at the foam excitation wavelength of 282 nm (4.40 eV energy), and only for low amounts of Mg doping^67^. We note however, that the beam depletion method relies on energy transfer from the photoexcited chromophore to the He droplet and is thus insensitive to species that are swiftly ejected upon photoexcitation, such as the Mg monomer, which is not present in the depletion spectra^67^. Taking into account that ${{{{\rm{Mg}}}}}_{n}^{+}$ cluster ions are ejected from the droplet for excitation of foam aggregates, but not for excitation of compact clusters (Fig. 4), might explain the low depletion signal observed for foam excitation. Furthermore, according to our global fit analysis the foam aggregate signal is 27% weaker than the dense cluster signal, which is in agreement with the weak beam depletion signal.

Our time-domain analysis provides additional insight by revealing the photodynamical response: Photoexcitation of the foam-like Mg_n_ configuration triggers nuclear dynamics leading to the contraction of the aggregate. This nanometer motion of Mg atoms is represented by significantly slower transient photoelectron signals, compared to the predominantly electronic dynamics of a compact van der Waals cluster. This difference in the transient response of the two Mg_n_ configurations is the essential ingredient for distinguishing the overlapping spectra through global fitting analysis. Purely based on frequency-domain information, this distinction cannot be made. The observation that both configurations are simultaneously present within the observed Mg_n_He_N_ ensemble is of relevance for cluster formation inside He droplets^24^. While the foam-like configuration is predicted to be favorable under steady-state conditions at the droplet temperature^33^, this weakly-bound aggregate can collapse during the pickup phase either by a hot (insufficiently cooled) Mg atom or when the dilute aggregate exceeds a critical size^43^. Cluster aggregation inside He_N_, in particular the formation of foam-like configurations, is thus governed by an interplay of kinetics and thermalization. Recent time-dependent simulations, following TD-DFT^68–71^, particle-based^69,70^, or hybrid^72^ approaches are able to account for such kinematic effects. These simulations reveal that the growth of compact clusters can be hindered by freezing in metastable configurations, with a certain probability depending on kinematic parameters.

Metastable separation through formation of a He barrier has been predicted, in addition to Mg^33^, for rare-gas^34,68–72^ and halogen^35^ atoms, as well as larger molecules^73^. Experimental evidence for the existence of a metastable configuration was reported in early deflection and mass spectrometric experiments of Ar, Kr, Xe, H_2_O and SF_6_, which find that the cross section for pickup is larger than that for coagulation^74^. Electronic spectroscopy of anthracene–Ar clusters in He_N_ revealed indications for the shielding of an attached Ar atom by a helium layer^75,76^. Very recently, electron diffraction also found evidence for large Xe-Xe distances with He located in between^71^. In experiments with bulk liquid He, a very similar stabilization of atoms was reported^77^. Cold impurity atoms introduced into the He solvent through a supersonic jet expansion are found to condensate in an ”impurity–helium solid”, characterized by a pronounced spatial separation of the impurities^78^. Such structures, investigated by means of optical spectroscopy, electron spin resonance and thermometry, were recently also observed for H_2_O clusters^79,80^.

While the ability of He_N_ to freeze aggregates in non-equilibrium structures has long been appreciated^81^, stable and well-defined large-distance separation of reactants through a solvent layer barrier provides fundamentally new perspectives for bond-formation studies. Helium droplets furthermore enable stable separation of surface-located and solvated species^28,31^ and the possibility to switch between the two locations through electronic excitation^29^ or through ionization, which recently enabled the real-time observation of the primary steps of ion solvation in helium^26^. Taking into account the formation of exciplexes, consisting of excited atoms and He^82–85^, shows that helium nanodroplet isolation holds great promise to study bond formation dynamics in various species. Such studies will provide insight into elementary processes accompanying the photoinduced formation of chemical bonds, such as the transient population of highly excited states above the excitation photon energy, as observed here for the formation of Mg clusters. The proposed energy pooling process relies on merging the energy of two or more excited Mg atoms to populate highly excited states. The ability of photon upconversion to trigger photoinduced processes that lie outside the available spectrum has implications in various fields, including photomedicine^86,87^. Efficient upconversion requires close distances of the involved particles, as recently demonstrated with solid-state organic chromophore blends^88^, whereas gas-phase configurations suffer from prohibitory low yields due to large interparticle distance^56,57^. The nanometer confinement provided by He droplets, together with flexible opportunities for generating tailor-made aggregates, thus provides a new and promising route to characterize the underlying energy and charge-transfer dynamics.

## Methods

Following a previous approach^37^, helium nanodroplets with a mean radius of 5.3 nm (13500 He atoms per droplet) are loaded with about ten Mg atoms (see Supplementary A for further details). Using an amplified Ti:sapphire laser (800 nm center wave length, 25 fs pulse duration), short pulses are generated and split into a pump and probe arm. The cross-correlation signal of the pump and probe pulse has a duration of (45 ± 3) fs. The pump pulse is tuned to 282 nm (4.40 eV photon energy) by an optical parametric amplifier, in order to trigger the collapse of the foam-like Mg_n_ aggregate through Mg 3^1^P_1_ ← 3^1^S_0_ excitation, which appears slightly blue-shifted in the aggregate^32^ relative to the bare atom transition^44^ (see Fig. 1a). The probe pulse at 404 nm (3.07 eV), obtained through frequency doubling, ionizes the system and photoelectron spectra are recorded with a magnetic bottle time-of-flight spectrometer. The time-resolved variation of these photoelectron spectra, recorded through variation of the pump–probe time delay, provides insight into the evolution of excited state populations. Applying a high voltage pulse to the repeller electrode about 100 ns after the laser pulses accelerates the remaining ions towards the detector and thus allows for a simultaneous detection of electrons and ions in each laser shot^89^. This procedure enables a statistical analysis of covariances between electron energy and ion species^45–47^.

## Supplementary information

Supplemental Material

## Data Availability

The data displayed in the Figures are available at Zenodo with the identifier 10.5281/zenodo.15363175, and are available from the corresponding author on reasonable request.

## Code availability

The code for the global fit analysis is available from the corresponding author on reasonable request.
