# Supplementary material for: Real-time tracking of energy flow in cluster formation
Source: Commun Chem. 2025 May 29;8:165. doi: 10.1038/s42004-025-01563-6 (PMC12119868; doi:10.1038/s42004-025-01563-6)
Supplement: Supplementary file 1 — Supplemental Material [file 42004_2025_1563_MOESM1_ESM.pdf]

Supplementary Information

Real-time tracking of energy flow in cluster formation

Michael Stadlhofer[1], Bernhard Thaler[1], Pascal Heim[1], Josef Tiggesbäumker[2,3], Markus Koch[1*]

*Corresponding author(s). E-mail(s): markus.koch@tugraz.at;

A Generation and doping of He droplets

We generate He droplets by expanding high-purity He through a 5 μm diameter nozzle at 20 bar pressure of and 11.6 K temperature. The droplet size follows a log-normal distribution with a mean number size of $\overline{N} = 13500$ [1]. The He droplets are doped with Mg atoms inside a resistively heated oven, where the number of Mg atoms entering the droplet can be controlled by changing the metal vapor pressure via the oven heating current. A quadrupole mass spectrometer is used to monitor the Mg pickup conditions. The Mg_n aggregate size limit can be estimated from the mass-to-charge spectrum in Fig. 3, where Mg_8^+ corresponds to the highest detected mass-to-charge ratio. By assuming that the parent cluster was split in half during pump–probe ionization, the largest initial cluster size can be estimated with Mg_{16}. To characterize the influence of the Mg doping level on bands (1)–(3) in the photoelectron spectrum (Fig. 2), the corresponding photoelectron signals are shown in Fig. A.1 as a function of the Mg oven heating current. While band (1) (2 to 3 eV binding energy) increases steadily with rising oven current, band (2) (0.5 to 2 eV binding energy) shows a local maximum at around 28 A. This opposing signal dependency for large doping levels supports the assignment of band (1) to compact Mg_n clusters and band (2) to a foam-like Mg_n configuration and is in line with the recent observation of spontaneous foam collapse for higher Mg doping levels [2]. PE band (3), associated with single Mg atoms (-0.5 to 0.5 eV binding energy) is most prevalent at lower oven currents, and is strongly suppressed for larger oven currents, confirming that the He droplets contain multiple Mg atoms for the applied heating current of 29 A.

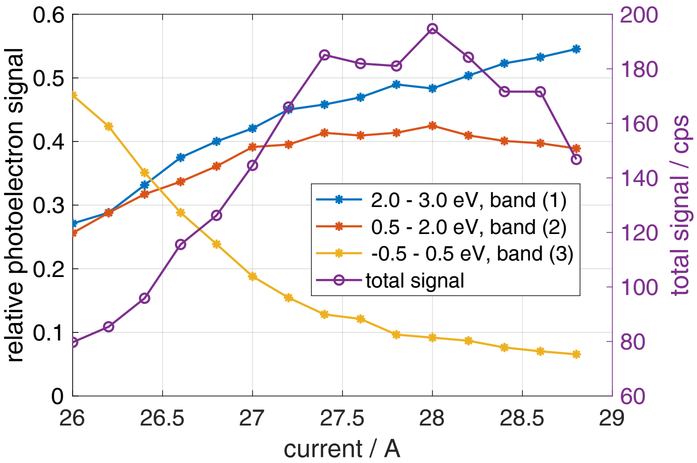

Fig. A.1 Dependence of the pump-probe photoelectron bands (c.f., Fig. 2) on the Mg doping level, represented as heating current applied to the Mg pickup oven. The photoelectron spectra for different heating currents are normalized and the depicted photoelectron signal is obtained through integration within the energy intervals given in the legend, and in time from -1 ps to 11 ps.

B Global fitting

For our global fitting analysis, the following two types of transient decay functions are used: (i) An instantaneously rising signal followed by exponential decay is modelled by a decay function of the form

$$N_i(t) = \frac{1}{2} e^{\frac{\sigma^2}{2\tau_i^2} - \frac{(t-t_0)}{\tau_i}} \operatorname{erfc}\left(-\frac{t-t_0}{\sqrt{2}\sigma} + \frac{\sigma}{\sqrt{2}\tau_i}\right), \tag{B.1}$$

which is obtained through convolution of a Gaussian function with a full halfwidth σ (representing the temporal instrument response function) centered at time zero, t_0, and a single exponential decay function with characteristic decay time τ_i [3]. In Eq. B.1 erfc is the complementary error function erfc(z) = 1 - erf(z).
(ii) Modeling of a delayed signal rise followed by an exponential decay is achieved through a sequential model, in which state A is excited by the pump pulse and decays with time constant τ_A into state B, which itself subsequentially decays with time constant τ_B. The corresponding decay function reads

$$N_i(t) = [N_A(t, \tau_A) - N_B(t, \tau_B)] \frac{\tau_A}{2(\tau_A - \tau_B)}, \tag{B.2}$$

where $N_A(t, \tau_A)$ and $N_B(t, \tau_B)$ are represented by Eq. B.1. The time constant τ_A thus corresponds to the characteristic rise time of the population $N_i(t)$, labeled τ_i^{rise} in the main text, whereas τ_B corresponds to the characteristic decay time of $N_i(t)$, labeled τ_i. In addition to the three DAS and corresponding decay functions discussed in the main text, in both energy regions an exponentially increasing background is considered by using Eq. B.2 with a corresponding rise time and a very long decay time

of 1 μs. The fitting procedure reveals that the background in the low binding energy region has a rise time of $\tau_{BG}^{low} = (3300 \pm 1200)$ fs and in the higher binding energy region has a rise time of $\tau_{BG}^{high} = (200 \pm 1200)$ fs.
There is still some interplay between DAS_3 and crosscorrelation in the low binding energy region, which suggests that N_3 might rise slightly slower than the single decay fit function allows with the temporal instrument response of $\sigma = 170$ fs. However, switching to a two-level decay fit function does not improve the fit for band (3). Figure B.1a summarizes all decay functions, and the corresponding decay associated spectra are shown in Fig. B.1b. In Fig. B.2 the quality of the fit result can be evaluated by comparing the measured time-resolved photoelectron spectrum in (a) to the global fit model in (b). The residuals in (c) show that the deviation between the two is below 10 % throughout the majority of the spectrum.

C Excitation probability for Mg atoms inside helium droplets

The energy pooling process outlined in the main text requires at least two excited Mg atoms inside a droplet. We therefore estimate the excitation probability of the Mg atoms for the applied laser parameters. The probability p_{abs} of a single photon from the pump laser pulse to be absorbed by one Mg atom is given by the ratio of absorption cross section σ_{abs} and the laser beam area A:

$$p_{\mathrm{abs}} = \frac{\sigma_{\mathrm{abs}}}{A} \tag{C.1}$$

Calculation of the absorption cross section σ_{abs}

Since the width of the absorption spectrum of Mg atoms solvated inside He droplets [4] is comparable to the spectral width of the short laser pulses, the absorption cross section σ_{abs} can be calculated by multiplying the frequency-integrated absorption cross section σ_0 with the overlap integral I_{overlap} of the line shape function of the broadened atom transition $g(\omega)$ and the spectral line shape of the laser pulse $\rho(\omega)$:

$$\sigma_{\mathrm{abs}} = \sigma_0 \times I_{\mathrm{overlap}} = \sigma_0 \int_{-\infty}^{\infty} g(\omega)\rho(\omega)\mathrm{d}\omega \tag{C.2}$$

Note that $g(\omega)$ and $\rho(\omega)$ are in units of s/rad. The frequency-integrated absorption cross section σ_0 for the Mg atom transition $3^1P_1 \leftarrow 3^1S_0$ follows from the Einstein A_{21} coefficient [5]:

$$\sigma_0 = \frac{1}{4}\frac{g_2}{g_1}\lambda^2 A_{21}$$

Here $g_2 = 3$ and $g_1 = 1$ are the degeneracies in the upper and lower level, respectively, and λ is the excitation wavelength. With the Einstein spontaneous emission rate for Mg of [6]

$$A_{21} = 4.91 \times 10^8 \mathrm{s}^{-1}$$

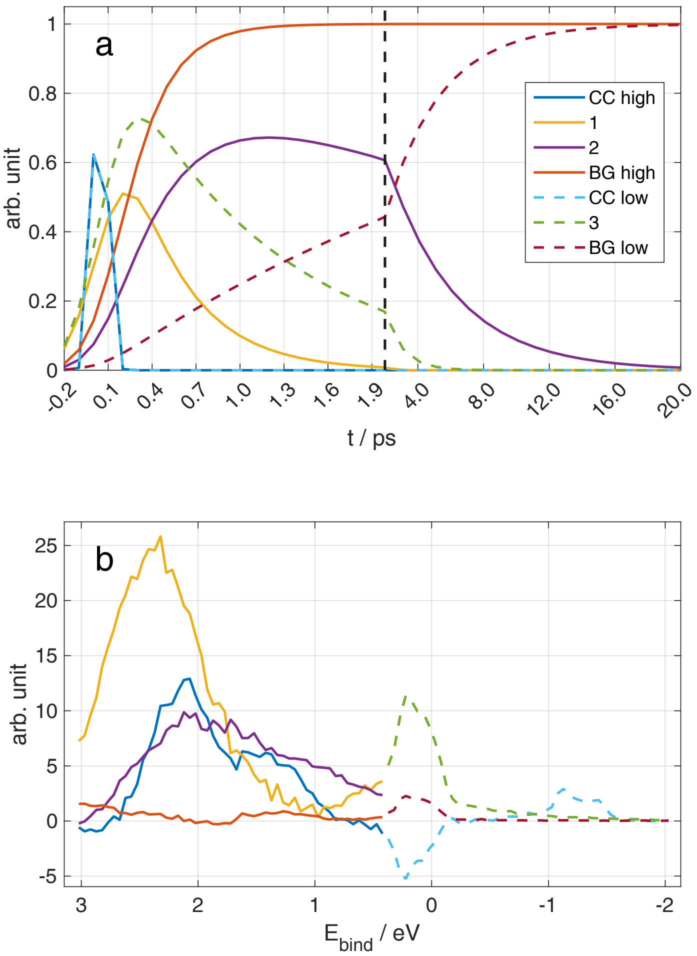

Fig. B.1 Overview of the global fit analysis. **(a)** Time-dependent decay functions for each decay associated spectrum. Note that the time-scale changes at 2 ps, indicated with the black-dashed vertical line. The crosscorrelation functions are abbreviated as CC, and the background functions as BG. Background and crosscorrelation also have the identifier high and low, which refers to the low or high binding energy region (see main text). **(b)** Decay associated spectra corresponding to the decay functions in (a).

and the $3^1P_1 \leftarrow 3^1S_0$ transition wavelength of Mg atoms in He droplets of [4]

$$\lambda = 282.5\,\text{nm},$$

we obtain the frequency-integrated absorption cross section σ_0 of

$$\sigma_0 = 2.90389 \times 10^{-5}\ \text{m}^2 \frac{\text{rad}}{\text{s}}$$

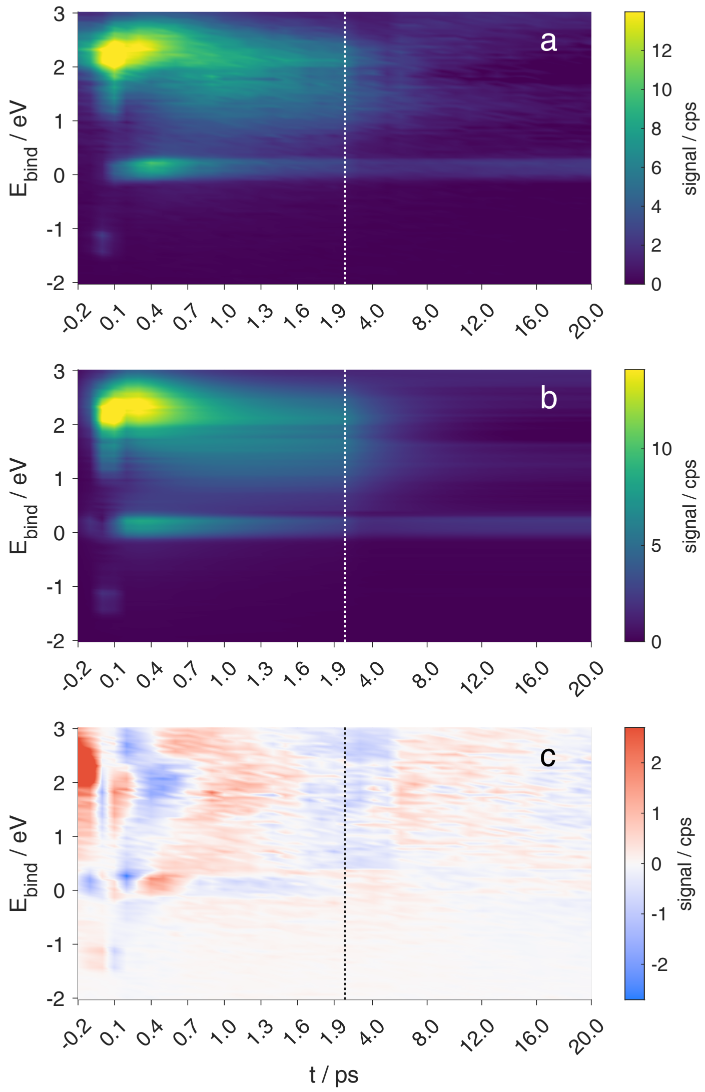

Fig. B.2 Evaluation of the global fit model. **(a)** Measured time-resolved photoelectron spectrum as depicted in Fig. 2a **(b)** Reconstruction of the time-resolved photoelectron spectrum, obtained by summing up all DAS contributions. **(c)** Residual plot obtained as difference of the measured (a) and reconstructed (b) spectra.

In using this value, we are assuming that the spontaneous emission rate for Mg does not change when the atoms are inside the helium droplet i.e. that the broadened lineshape is not caused by a shorter lifetime of the excited state.
For the spectral line shape of the laser pulse $\rho(\omega)$, a Gaussian spectrum with standard

deviation s_{laser} centered at the mean angular frequency ω_0 is assumed:

$$\rho(\omega) = \frac{1}{\sqrt{2\pi} s_{\text{laser}}} e^{-(\omega-\omega_0)^2/(2s_{\text{laser}}^2)}$$

From the measured spectrum of the pump pulses, with a full width at half maximum (FWHM) bandwidth of $\Delta\rho = 3.5$ nm centered at 282.5 nm, the standard deviation of the laser spectrum in units of angular frequency can be calculated:

$$s_{\text{laser}} = 3.51 \times 10^{13}\,\text{rad/s}$$

For the absorption spectrum of Mg in helium droplets, we also assume a Gaussian line shape with a FWHM of $\Delta g = 4$ nm, [4] yielding a standard deviation of the line shape function of

$$s_{\text{abs}} = 4.01 \times 10^{13}\,\text{rad/s}.$$

With the spectral distributions of the laser and absorption line, we can calculate the overlap integral:

$$I_{\text{overlap}} = 7.488 \times 10^{-15}\,\text{s/rad}$$

The final cross section according to Eq. (C.2) is then

$$\sigma_{\text{abs}} = 2200 \text{ Mb}.$$

Calculation of the laser beam area.
For a Gaussian beam, the area $A(z)$ and diameter $d(z)$ as function of the distance z to the beam waist are given by the following relations [7]:

$$A(z) = \pi \left(\frac{d(z)}{2}\right)^2$$

$$d(z) = d_0 \sqrt{1 + \left(\frac{z}{z_R}\right)^2}$$

$$z_R = \frac{\pi d_0^2}{4\lambda}$$

$$d_0 = \frac{4\lambda f M^2}{\pi D},$$

where f is the focal length of the lens, λ is the laser wavelength, M^2 is the beam quality and D is the diameter of the laser beam at the lens. With the laser beam parameters listed in Tab. II, we obtain the following value for the laser focus area:

$$A = 4.63 \times 10^{14} \text{ Mb}$$

Table C.1 Laser beam parameters: $E_{\text{pulse}} \ldots$ pulse energy, $D \ldots$ diameter of the laser beam at the lens, $z \ldots$ distance to the beam waist, $f \ldots$ focal length of the lens, $M^2 \ldots$ beam quality factor.

E_{pulse} / μJ	D / mm	z / mm	f / mm	M^2
1	2	0	1000	1.35

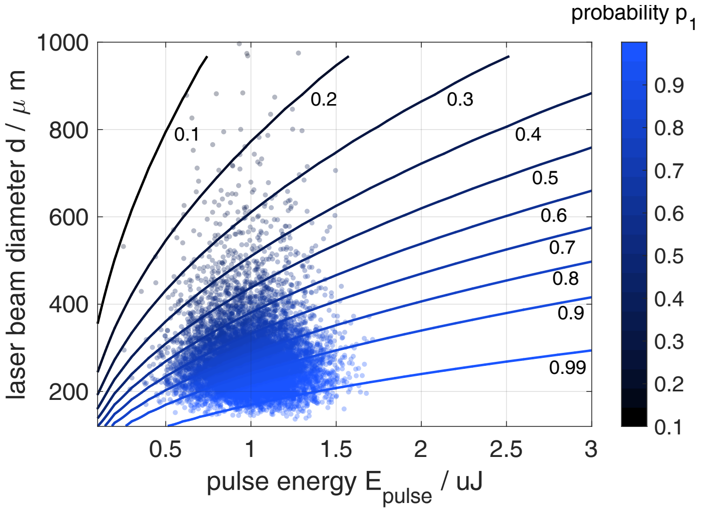

Fig. C.1 Sampled probabilities p_1, for one Mg atom to be excited by N photons. The contours and colors are used to indicate the excitation probability for a given laser beam diameter d and pulse energy E_{pulse}.

Calculation of the excitation probability.
The single photon absorption probability of a Mg atom inside a He droplet, calculated according to Eq. (C.1), is:

$$p_{\text{abs}} = \frac{2.2 \times 10^3 \text{ Mb}}{4.63 \times 10^{14} \text{ Mb}} = 4.75 \times 10^{-12}$$

For the N photons contained in a laser pulse, the excitation process can be modeled by a Bernoulli trial, i.e., the probability that at least one photon excites the atom follows a Bernoulli distribution:

$$p_1 = \sum_{n=0}^{N-1} p_{\text{abs}} \times (1 - p_{\text{abs}})^n = 1 - (1 - p_{\text{abs}})^N \quad \text{(C.3)}$$

Table C.2 Mean and standard deviation for the parameters used in random sampling: $E_{\text{pulse}} \ldots$ pulse energy, $D \ldots$ laser beam diameter at the focusing lens, $z \ldots$ sample position with respect to the laser beam waist.

	E_{pulse} / μJ	D/ mm	z / mm
mean	1.0	2.0	0
standard deviation	0.2	0.5	50

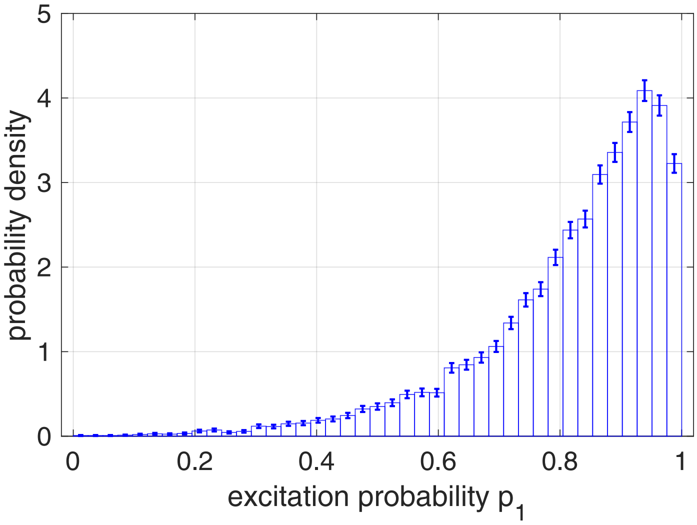

Fig. C.2 Numerical probability density for the excitation probability p_1, given the uncertainty in pulse energy, beam diameter at the lens and distance from sample in Table C.2.

The number of photons N in the pulse can be calculated from the pulse energy E_{pulse} and photon energy ($\hbar\omega_0$ neglecting the bandwidth of the laser):

$$N = \frac{E_{\text{pulse}}}{\hbar\omega_0}$$

For the value of $p_{\text{abs}} = 4.75 \times 10^{-12}$, pulse energy of 1 μJ and photon energy of $h \times 1.061 \cdot 10^{15}$ Hz, Eq. (C.3) yields a pump-pulse excitation probability of a single Mg atom inside a He droplet of

$$p_1 = 0.9988.$$

Since this value is derived for optimal laser beam parameters, which might deviate in the experiment, we assume a Gaussian distribution of the parameters E_{pulse}, D and z (see Tab. C.2) and apply statistical sampling to generate a distribution for the excitation probability, from which we determine the mean and standard deviation. Large uncertainties of the parameters are chosen in order to obtain a robust estimate for the excitation probability. For each set of random sample parameters, an excitation probability p_1 is calculated. In Fig. C.1, the pulse energy E_{pulse} and laser beam diameter

d (at the sample) are chosen to depict the dependency of the excitation probability in a 2D plot, together with the contour lines of the $p_1(d, E_{\text{pulse}})$ function.
Fig. C.2 shows a histogram of the sampled probabilities p_1, which can be interpreted as a probability density for the excitation probability p_1, resulting from the distributions of input parameters. The numerical mean and standard deviation of this distribution are $p_1 = 0.81$ and $\sigma_{p_1} = 0.15$, showing that Mg atom excitation inside He droplets is very likely for a broad range of experimental laser parameters. The high single-atom excitation probability makes excitation of multiple atoms inside a single droplet plausible.

D Energy level diagram

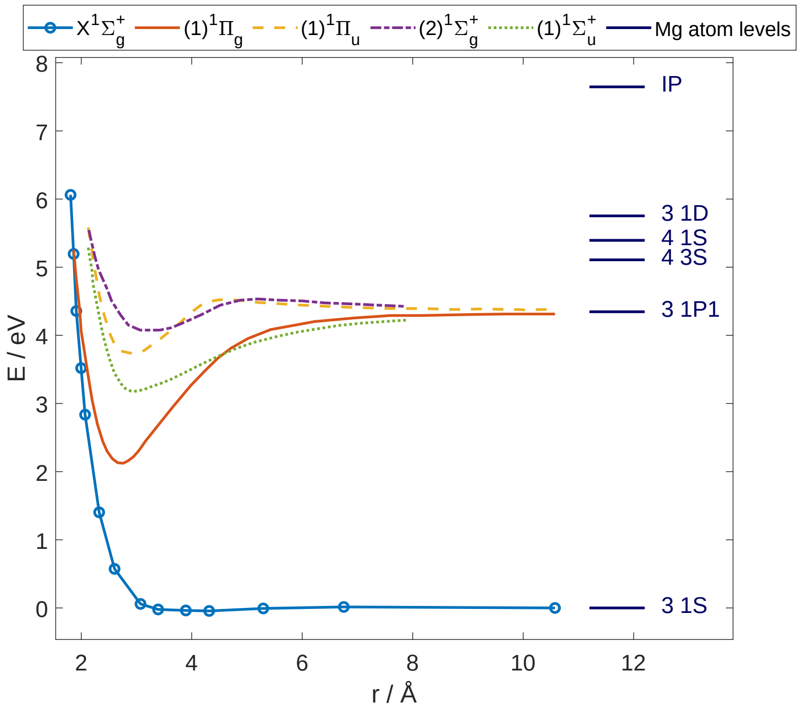

Fig. D.1 Energy level diagram showing the ground state and relevant excited states of the Mg dimer, as well as selected Mg atom energy levels and the atom ionization potential [8]. The potentials are reproduced from Ref. [9].

References

[1] Toennies, J.P., Vilesov, A.F.: Superfluid helium droplets: a uniquely cold nanomatrix for molecules and molecular complexes. Angew. Chem. Int. Ed. **43**(20), 2622–2648 (2004) https://doi.org/10.1002/anie.200300611

[2] Kazak, L., Meiwes-Broer, K.-H., Tiggesbäumker, J.: Ionization potentials of Mg_N (N = 7–56) clusters formed by spontaneous collapse of magnesium foam in helium nanodroplets. Physical Chemistry Chemical Physics **24**(38), 23350–23356 (2022) https://doi.org/10.1039/d2cp03075f

[3] Stokkum, I.H.M., Larsen, D.S., Grondelle, R.: Global and target analysis of time-resolved spectra. Biochimica et Biophysica Acta (BBA) - Bioenergetics **1657**(2-3), 82–104 (2004) https://doi.org/10.1016/j.bbabio.2004.04.011

[4] Przystawik, A., Göde, S., Döppner, T., Tiggesbäumker, J., Meiwes-Broer, K.-H.: Light-induced collapse of metastable magnesium complexes formed in helium nanodroplets. Physical Review A **78**(2) (2008) https://doi.org/10.1103/PhysRevA.78.021202

[5] Hilborn, R.C.: Einstein coefficients, cross sections, dipole moments, and all that. American Journal of Physics **50**(11), 982–986 (1982) https://doi.org/10.1119/1.12937

[6] Kelleher, D.E., Podobedova, L.I.: Atomic transition probabilities of sodium and magnesium. a critical compilation. Journal of Physical and Chemical Reference Data **37**(1), 267–706 (2008) https://doi.org/10.1063/1.2735328

[7] Saleh, B.E.A., Teich, M.C.: Fundamentals of Photonics (Wiley Series in Pure and Applied Optics), p. 77. Wiley-Interscience, ??? (2007)

[8] Kramida, A., Yu. Ralchenko, Reader, J., and NIST ASD Team NIST Atomic Spectra Database (ver. 5.10), [Online]. Available: `https://physics.nist.gov/asd` [2023, August 11]. National Institute of Standards and Technology, Gaithersburg, MD. (2022)

[9] Amaran, S., Kosloff, R., Tomza, M., Skomorowski, W., Pawłowski, F., Moszynski, R., Rybak, L., Levin, L., Amitay, Z., Berglund, J.M., Reich, D.M., Koch, C.P.: Femtosecond two-photon photoassociation of hot magnesium atoms: a quantum dynamical study using thermal random phase wavefunctions. The Journal of Chemical Physics **139**(16), 164124 (2013) https://doi.org/10.1063/1.4826350
